# Tuber yield and water efficiency of early potato varieties (*Solanum tuberosum* L.) cultivated under various irrigation levels

**DOI:** 10.1038/s41598-021-97899-9

**Published:** 2021-09-27

**Authors:** Anna Jama-Rodzenska, Grzegorz Janik, Amadeusz Walczak, Katarzyna Adamczewska-Sowinska, Jozef Sowinski

**Affiliations:** 1 Institute of Agroecology and Plant Production, Wroclaw University of Environmental and Life Sciences, Grunwaldzki Square 24A, 50-363 Wroclaw, Poland; 2 Department of Environmental Protection and Development, Wroclaw University of Environmental and Life Sciences, Grunwaldzki Square 24, 50-363 Wroclaw, Poland; 3 Department of Horticulture, Wroclaw University of Environmental and Life Sciences, Grunwaldzki Square 24A, 50-363 Wroclaw, Poland

**Keywords:** Agroecology, Climate-change ecology

## Abstract

This study aims to determine the effects of differences in variety and irrigations levels on potato yield (appropriate humidity, temperature, shading the plants from the sun if necessary) on potato yield in greenhouse conditions. Functions of potato production with respect to water use were developed for five dates of measurements of plant growth, in relation to the various vegetation phases. On the basis of potato vegetation phases, the potato water demand was determined. An experiment was conducted using the randomized sub-block method. The first order factor were the two varieties of potato that were grown under drip irrigation with three water regimes as a second factor experiment: level 1 (pF 2.7), level 2 (pF 2.5) and level 3 (pF 2.2). The variety had a significant effect on the weight of potato tubers. The irrigation level had only a significant effect on the total potato biomass. The potato harvest date had a significant effect on both of the examined treatments. The growth dynamics of the aboveground part and potato tubers were the highest in conditions of constant level 1. Regardless of the variety studied, on level 3 caused a reduction in potato biomass production.The highest water consumption was observed during the tuber potato growth period to flowering. The values were varied from 0.39 l/pot day level 1 (in both investigated cultivars) to 0.99 l/ pot/day (humidity level 3 for Julinka cultivar) in July. The most intensive increase in water consumption was observed at the level 3. The average W index of the average daily water dose calculated for Denar cultivar amounted 0.40 l day^−1^ in the first period (O1) to 0.79 l day^−1^ in the fifth period (O5) and for Julinka cultivar 0.49 l day^−1^ (O1) to 0.92 l day^−1^ (O5). Stress due to water shortage and/or excessive levels of water in the soil negatively influenced the yield of potato tuber. Potato varieties reacted differently to soil water content.

## Introduction

Potato is one of the most important agricultural crops grown in almost all world. Potato tubers are used for food, fodder, industrial and seed purposes^1–3^ and its cultivation area in developing countries is showing an upward trend, especially in Africa and Asia. Water shortage and exscessive may negatively impact on the yield and quality of potato, by obtaining small, medium or dimnishing sized tubers which may be subject to to decay or extending the period from germination to emergence. In order to prevent these changes, the farmers are searched primarily benefit from biological progress and select varieties with high and stable productivity well adapted to a changing climate and water stress conditions connected with deepening drought periods and biotic factors (pest, diseases)^4–6^.

Potato has high climate requirements. The availability and amount of water at specific growing stages effect the potato quality and yield^1,7,8^. Potato is a water-stress crop, and a long-term lack of water is the main abiotic factor that limits yield^7,8^. Even a small water deficit can significantly reduce tuber formation, deformation and quality deterioration, and thus reduce potato production efficiency. Potato tuber yield losses in the absence of water can reach as much as 69%^4^, and also reduces the development of the aboveground part: leaf and stem biomass. The pot experiment conducted on the dynamics of the aboveground growth parts under the excess water also shows large differences between varieties^1–3,9^. Tuber yield is closely related to soil humidity and genetic properties. Some species and potato varieties produce higher yields when water is limited^10,11^.

The varietal differences result from the different lengths of the growing period, and with a short vegetation, the greatest demand for water occurs in the early growing stages^12^. The critical periods of potato demand for water recognized in the literature are: flower bud formation—flowering—tuber formation (tuberization)—ripinning of the plants (tuber mass accumulation)^9,13,14^. Milić et al. (2010)^15^ stated that the optimum soil humidity for potato is 70% of the field water capacity when cultivated on medium textured soil. Begum et al. (2018)^16^ stated that potato should be provided with adequate humidity during the formation of stolons and the initial development of tubers. Water management should be specificly adjusted to the potato varieties and avoid excessive moisture or water (lack of water) stress^1,4,17^. Shortage of appropriate soil water during tuber development can cause tuber deformation, while too much soil water can exacerbate root and tuber root diseases^18^.

Currently as a result of climate change, and uneven rainfall distribution and fluctuations, the risk of water stress in this species is greater^6,19,20^. Scenarios investigating the consequences of climate changes by 2055 and predict a global reduction in potato tuber production (by 2–6%). Larger decreases will possibly be observed in subsequent years: up to 2085 (by 2–26%)^21^. Such effects of climate change will be particularly noticeable in the warmer regions covering most developing countries and yield decreasing by 20–40% in the future^19^.

The present research sought to verify the hypothesis that potato varieties have different reactions to permanent soil water regimes. The research tested the effect of optimal, excess and limited soil humidity on potato growth dynamics. Optimal or stressed soil water regime influenced on water use efficiency and potato production.

Recognition of these issues is the key to saving water in the production of early potato varieties. It could allow irrigation treatments to be carried out on a larger area.

## Materials and methods

### Experimental setup and design

**Figure 1:**
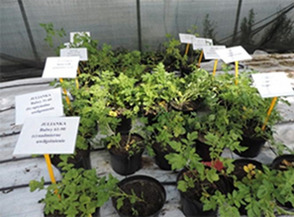
Establishment of pot experiment and an irrigation system with irrigation line and pot drippers.

A pot experiment under controlled conditions (temperature, humidity) in greenhouse was carried out in 2018 (during the growing season May to July) at the Research and Didactic Station in Psary, part of the Department of Horticulture at Wroclaw University of Environmental and Life Sciences (Fig. 1). Research was carried out in plastic pots with a capacity of 11 L. Mineral soil—light sandy loam—with an optimal content of nutrients necessary for proper potato development. Chemical and physical properties of the experimental field soil related to irrigation are presenteted in Jama-Rodzeńska et al. (2020)^22^. Selected, potato tubers *Solanum tuberosum* L. (Denar and Julinka variety) were planted manually (5 cm from the pot's surface) on 5th May, 2018, and harvested five times on different growing stages finally on 18th of July 2018 (the last date of harvest). Potato harvest was conducted at: O1—beginning of tuber growth (BBCH 40/400), O2—beginning of flowering (BBCH 52/502), O3—flowering (BBCH 60/600), O4—flower falling (BBCH 69/609), and O5—yellowing of leaves (BBCH 91/91). The harvest was carried out on: 29/05, 13/06, 20/06, 04/07 and 18/07/2018. Each treatment was repeated 6 times (6 pots). No fertilizers treatment were applicated.

The pot experiment was designed in a randomized sub-block system. The first factor comprised potato varieties: Denar—very early edible, salad type; and Julinka—early type of multiuse type. The second factor was the soil humidity level depending on varying amounts of water supplied: water deficit (pF 2.7—level 1), optimal water level (pF 2.5—level 2), and excess water (pF 2.2—level 3). The Denar variety is more drought tolerant. The Julinka variety has higher water and soil requirements. In total, experiments were carried out in 180 pots. The height of the pot was 20 cm and diameter 14 cm. The experiment was conducted on degraded chernozem soil, Gleyic Calcic Chernozem soil (Food Agriculture Organization–World References Base, FAO–WRB). Soil mineral particle parts size structure corresponded to sandy clay^23^. The soil chemical composition used for the pot experiment was as follows: pH—7.16, salinity 155 µS/cm, P, K, Ca, Mg, nitrates (in mg/dm^3^): 51, 52, 420, 20, 1.31, respectively^23^.

### Establishment of pot experiment and an irrigation system with irrigation line and pot drippers

**Figure 2:**
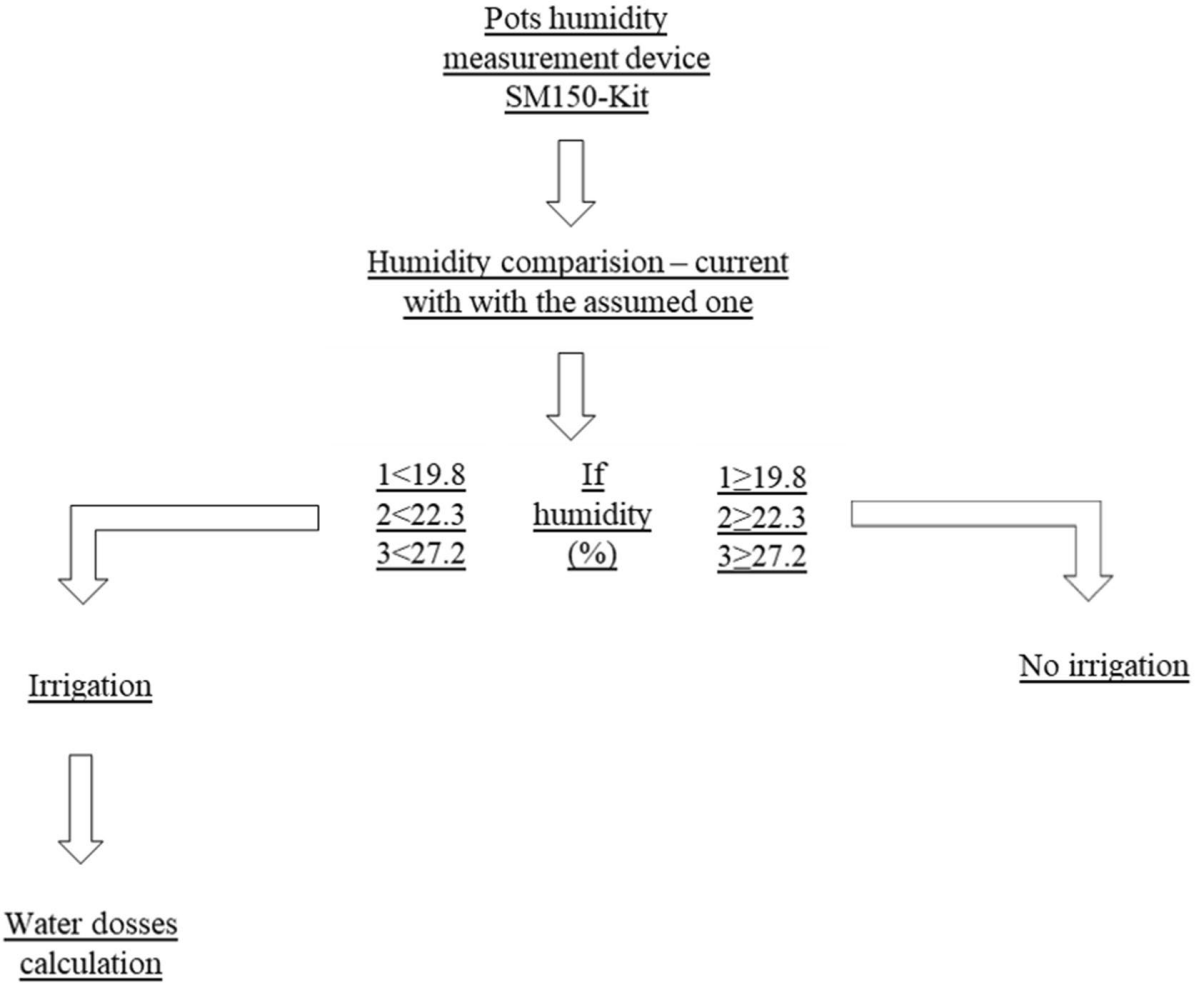
Procedural diagram for determining water irrigation doses.

Irrigation doses were determined on the basis of specific pF values for the soil used in the experiment^22^. Doses of tap water were given to the plants regularly and automatically by means of a drip line with a water dripper, into each pot. Drippers delivered water to the soil on rate of 2 l/h, with low, compensation pressure (4 bar). Irrigation was adjusted using a timer to maintain a specific soil humidity level and dose-specific amount of water. Doses of water were determined twice a week and based on soil humidity measurements made with an SM150-Kit capacitive probe with an automated reader. The soil moisture content of the top 30 cm was measured by the gravimetric method. The amount of soil water in the 30 cm top layer was used to initiate irrigation treatment. Irrigation treatments were established to refill a 30 cm depth-rooting zone. The amount of water used for irrigation was monitored in line with soil humidity measurements made at 3/4-day intervals. The amount of water used was regulated by the electronical controller and time of water application and water dosses calculated to the assumed values and the current humidity level, using the following procedure (Fig. 2). The amount of water used was recorded after each irrigation cycle and summing.

### Biometric measurements

On each harvest date, biometric measurements of plants were performed. Measurements were conducted for six plants in each variant and for 36 plants on each harvest date. After removal from pots, the plants were washed and dried, and then the mass of the crop biomass part was determined. Representative samples were taken to specify the biometric characteristics of the aboveground and underground parts. The weight of whole plants, stems with leaves, roots with stolons and tubers were assessed. A laboratory balance with an accuracy of 0.1 g was used to assess the parameters.

### Water use efficiency

Analysis was performed of the efficiency of water consumption based on the amount of its use during irrigation on individual days in terms of weight of tubers (g) and weight of whole plant biomass (g).

The following water consumption efficiency indicators were calculated:

1 $$ W = \frac{A}{E}. $$

W is the average water consumption for varieties (l day^−1^), soil humidity level (1), (2), (3), and periods—(O1), (O2), (O3), (O4), (O5), A is the water consumption within potato growing stages, E is the number of days in growing stages.

2 $$ W1 = \frac{F}{J}. $$

W1 is the average water consumption for varieties (l day^−1^), soil humidity level (1), (2), (3) from tuber planting, F is the water consumption from potato planting date, J is the number of days from planting.

3 $$ W2 = \frac{D}{E}. $$

W2 is the average daily potato tuber mass increases (g day^−1^) depending on varieties, soil humidity levels (1), (2), (3) in each of five potato growing stages (O1), (O2), (O3), (O4), (O5), D is the potato tuber biomass increases within growing period, E is the number of days in period.

4 $$ W3 = \frac{I}{J}. $$

W3 is the average daily potato tuber mass increase (g day^−1^) depending on varieties, soil humidity levels (1), (2), (3), I is the potato tuber biomass increases from planting, J is the number of days from planting.

5 $$ W4 = \frac{A}{B}. $$

W4 is the ratio of average daily water consumption to average daily tuber mass increase (l g^−1^) dependent on soil humidity levels (1), (2), (3), in each of five potato growing stages (O1), (O2), (O3), (O4), (O5), A is the average water consumption within potato growing stages, B is the potato tuber mass increase within growing stages.

6 $$ W5 = \frac{F}{G}. $$

W5 is the ratio of average daily water consumption to average daily tuber mass increase (l g^−1^) dependent on soil humidity levels (1), (2), (3), F is the water consumption from potato planting date, G is the average daily potato tuber mass increase from planting.

### Statistical analysis

The results of potato biometric measurements were subjected to Anova/Manova statistical analysis in Statistica software (version 13.1 StatSoft, Poland). Two or three-factor variance analysis (ANOVA) was performed. Mean values were compared on the basis of Fisher's test at the significance level α = 0.05.

Excel, PowerPoint and Statistica were used to prepare figures. Pursuant to the empirical data collected at specific harvesting dates, trend lines representing a change in the structure of potato biomass were determined, depending on the variety and humidity level.

Water consumption indicators (W, W1, W2, W3, W4, W5) were calculated on the basis of correlation analysis and simple directional coefficients approximating empirical data. Results are presented in the form of linear functions, taking into account the correlation coefficients R^2^.

### Research involving plants

Experimental research and field studies on potato plants were complied with relevant institutional guidelines.

## Results and discussion

### Water use

Many potato physiological features (photosynthesis intensity, leaf water potential) morphological and agronomic features as the Soil Plant Analysis Development (SPAD) and dry matter content can be used as indicators of potato water stress. In this result water consumption and the average daily amount of water used for irrigation differed over the growing season, but differences also occurred between varieties and the humidity level (Table 1). When irrigating the Julinka variety at all stages of the growing season, regardless of the established pF values, water consumption per pot was higher. The average dose of water supplied per pot was 9.7%, 30.7% and 26.6% greater than for the Denar variety, at humidity levels 1, 2 and 3, respectively. The highest water consumption was observed during the potato growth period from BBCH 40/400 to 69/609 and ranged from 0.39 l/pot /day (level 1) to 0.99 l/ pot/day (level 3).

**Table 1:** Water consumption per pot within potato growing stages (in liters) and average consumption of water per pot (in brackets).

Potato growing periods^a^	Denar			Julinka		
	Humidity level			Humidity level		
	1st (pF 2.7)	2nd (pF 2.5)	3rd (pF 2.2)	1st (pF 2.7)	2nd (pF 2.5)	3rd (pF 2.2)
I	5.90 (0.26)	7.45 (0.32)	9.60 (0.42)	6.18 (0.27)	8.03 (0.35)	11.35 (0.49)
II	3.30 (0.22)	4.80 (0.32)	6.50 (0.43)	4.02 (0.27)	5.42 (0.36)	8.50 (0.57)
III	2.00 (0.29)	2.70 (0.39)	3.90 (0.56)	2.70 (0.39)	4.10 (0.59)	5.90 (0.84)
IV	3.30 (0.24)	4.70 (0.34)	8.70 (0.62)	3.50 (0.25)	7.50 (0.54)	11.50 (0.82)
V	5.10 (0.39)	6.40 (0.49)	10.90 (0.84)	5.10 (0.39)	9.00 (0.69)	12.90 (0.99)
Total from planting to 5th harvest	19.60	26.05	39.60	21.50	34.05	50.15

^a^I—from tuber planting to BBCH 40/400, II—from BBCH 40/400 to 52/502, III—from BBCH 52/502 to BBCH 60/600, IV—from BBCH 60/600 to BBCH 69/609, V—from BBCH 69/609 to BBCH 91/901.

The highest water consumption in both potato varieties occurred in July (11–18 July). Analyzing the remaining two months of the irrigation period, it can be seen that in June the plants used less water than in July. Seasonal irrigation doses in mid-early potato of studies of Rolbiecki et al. (2015)^9^ ranged from 40 to 170 mm, and the highest daily values of field water consumption (over 3 mm) occurred in July, similar to the results in this research.

Depending on the irrigation system, water consumption efficiency in potato varies from 5.4 to 12 kg m^−3^^16,24^. Drip irrigation is one of the most effective methods and ranged from 6.3 to 8.6 kg m^−3^ (Sharma 2007)^25^. Different values for average WUE index’ in potato cultivation were obtained by Ati et al. (2012)^26^, and indicated value ranged from 5.9 to 12.2 kg m^−3^. In present research, average WUE index’ for the Denar variety was from 0.00 l day^−1^ in the 1st period to 0.79 l day^−1^ in the 5th harvest period, while for the Julinka it was from 0.49 to 0.92 l day^−1^, respectively.

In the research by Zin El Abedin et al. (2019)^27^ the amount of water used for irrigating potato amounted to 1505 mm and 1062 mm for FI (full irrigation) and PRD (partial root zone drying) variants, respectively. The use of 50% of water consumption in the PRD reduced water productivity (WP), as compared to water stress in the form of excess FI and deficit irrigation (DI). A large amount of water in conditions of water deficit causes losses due to evaporation and leads to degradation of the soil environment. In turn, in this research the highest water consumption in both varieties was found at level 3, 39.60 l for the Denar variety and 50.15 l for the Julinka variety.

Pszczółkowski et al. (2009)^28^ showed that early potato varieties water requirements in the period from May 1 to August 31 amounted to 336.4 mm, with greatest requirements in July (108—119.6 mm). In our research, the amount of water used depended on the assumed humidity level and amounted from 19.60 × 10^3^ to 39.60 × 10^3^ cm^3^ for the Denar variety and between 21.50×10^3^ to 50.15 × 10^3^ cm^3^ for the Julinka (Table 1).

### Total potato and tuber mass

The total weight of plants aboveground—(stems with leaves) and underground (tubers, stolons and roots) was greater in water humidity level 1 than in humidity levels 2 and 3 (Table 2). Administration of increased amounts of water in the later stages of potato growth resulted in inhibition of biomass growth, mainly for the Julinka variety. At the 5th harvest time, at humidity level 3, the total weight and the weight of tubers were 59.2% and 54.7% lower than those obtained at level 1, respectively. At the same time, the difference for Denar was 11.9% and 18.8%, respectively. Begum et al. (2015, 2018)^16,22^ and Reyes-Cabrera et al. (2016)^5^ showed that the production of total and commercial tuber yield was strongly dependent on the total biomass production and its structure.

**Table 2:** Potato total biomass and tuber increase depending on water humidity level (g per plant).

Potato growing periods^a^	Potato biomass—total (g)		Tuber mass (g)	
	Denar	Julinka	Denar	Julinka
**Humidity level 1**				
I	74.7	82.6	0.0	14.2
II	169.7	201.8	16.7	35.8
III	203.7	241.0	28.8	52.7
IV	294.0	393.3	121.3	146.8
V	395.6	598.7	196.7	303.6
**Humidity level 2**				
I	47.3	94.2	6.0	10.0
II	127.9	152.7	10.8	20.0
III	159.7	210.0	24.3	61.1
IV	237.3	330.5	84.7	171.1
V	304.9	393.5	143.5	229.4
**Humidity level 3**				
I	60.4	69.1	0.0	11.7
II	171.5	119.5	17.5	38.3
III	195.8	139.9	36.6	51.1
IV	244.9	193.2	91.5	121.6
V	348.7	244.3	159.8	137.5

^a^Explanation under Table 1.

A three-factor analysis of variance showed that the total weight as well as the weight of potato tubers differed significantly by the humidity level and the variety. A significant effect was found for humidity level on the total weight and tuber weight for the Denar variety and tuber weight for the Julinka variety (Table 3).

**Table 3:** Variance analysis for total biomass and tuber of potato depending on factors (significance verified by the Fisher test).

Factor	Sum of squares	df	Mean square	F	Significance
**Potato total biomass**					
Denar × H × T	826,240.8	14	59,017.20	16.799	< 0.001***
Julinka × H × T	1,484,028.0	14	106,002.0	9.845	< 0.001***
Denar × H	41,285.6	2	20,642.80	1.713	0.186
Julinka × H	339,954.4	2	169,977.2	7.578	< 0.001***
Denar × T	765,228.8	4	191,307.2	50.111	< 0.001***
Julinka × T	986,073.9	4	246,518.5	16.051	< 0.001***
Varieties	34,800.7	1	34,800.74	1.832	0.178
H	126,298.0	2	63,148.99	3.398	0.036*
T	1,743,185.0	4	435,796.4	45.589	< 0.001***
**Potato tuber biomass**					
Denar × H × T	292,917.8	14	20,922.7	11.377	< 0.001***
Julinka × H × T	495,915.8	14	35,422.6	8.550	< 0.001*
Denar × H	5516.635	2	2758.3	0.564	0.5709
Julinka × H	23,281.82	2	11,640.9	1.293	0.280
Denar × T	280,901.7	4	70,225.4	39.810	< 0.001*
Julinka × T	423,364.4	4	105,841.1	23.473	< 0.001***
Varieties	43,771.1	1	43,771.1	6.296	0.0113*
H	12,241.3	2	6120.7	0.854	0.428
T	696,366.4	4	174,091.6	52.089	< 0.001***

*d.f. * degree freedom, *F* Fisher-Snedecor distribution, *Denar* potato varieties, *Julinka* potato varieties, *H* humidity level (1. 2, 3), *T* potato harvest term (I, II, III, IV, V).

*,**,***Significance at p < 0.05, p < 0.01 and p < 0.001.

Analysis of variance showed a significant impact of the variety on potato plant weight, while it did not show significant interaction of weight and weight of tubers between measurement dates. No significant effect was obtained for interaction between the factors studied (Table 3).

Wang et al. (2009)^29^, concluded that the use of irrigation significantly contributed to an increase total and commercial tubers of medium-early Folva variety yield and its quality. Ossowski et al. (2013)^30^, shown that irrigation had a significant effect on medium-early potato varieties: Barycz, Mors, Triada tuber yield. When using drip irrigation, yield increased by 26%. In turn, Mazurczyk et al. (2007)^31^ showed that drip irrigation increased the tuber yield from 29.4–37.5 to 45.1–54.4 t·ha^−1^.

Over the period from the 1st to the 5th harvest date, the total plant biomass increased from 3.5-fold (Julinka—level 3) to 7.2-fold (Julinka—level 1). On the first harvest, Denar did not produce tubers at levels 1 and 3, and for level 2 its weight was the lowest (6 g from a pot). The increase in tuber weight to the last harvest date was the highest for level 2: 23.9- and 22.9-fold, in Denar and Julinka varieties, respectively. At level 3, the growth dynamics of tubers was the lowest: 11.7 times for the Julinka and 9.1 times for the Denar variety (measured from the second harvest date). The highest total biomass increases and tuber weight was found between the 3rd, 4th and 5th dates when humidity was at levels 1 and 2, and between the 3rd and 4th dates at level 3.

Kumari et al. (2011, 2018)^1,2^ concluded that drip irrigation significantly contributed to an increase in potato tuber yield 18% greater than with other irrigation methods. Xu et al. (2010)^32^ achieved higher yields using the same irrigation system (40–48 t ha^−1^), and potato tuber weight was reduced under the slight water stress. Potato reacts to stress when soil water tension exceeds 20 kPa^24^. In a study by Amer et al. (2016)^33^ potato tuber yield also decreased with the application of excessive irrigation, resulting in greater stress, increased vegetative growth and potential leaching of nutrients from the root zone.

Changes also occur in the quality of potato tubers, such as the shape, skin smoothness and chemical composition^34^.In the research carried out by Zin El-Abedin et al. (2019)^27^ differences were found in potato tuber yield depending on the irrigation variant. At FI, the highest tuber yields of 31.77–35.91 Mg ha^−1^ were obtained. Water deficiency reduced tuber yield, in DI variants, by 53.24–65.15% as compared to the FI. Similar results were obtained by Kumari et al. (2011)^1^. In the present research, the tuber weight of the Denar variety in the fifth term in level 1, increased by 26% compared to the irrigation at level 2 and was a 24% increase for the Julinka variety under similar conditions. At humidity level 3 there was a decrease in total biomass by 12% and 59% (for Denar and Julinka, respectively) in comparison obtained at level 1. In the research Liu et al. (2006)^35^ the aboveground biomass reached the highest values in excess water conditions.

Potato varieties react differently to the humidity of the soil. Mahmood et al. (2016)^36^ response of potato varieties diversity to soil water deficit, also Hassanapanah (2010)^17^ showed the reaction of potato varieties to stress conditions. In our study, a higher total and tubers weight was found for the Julinka variety than for the Denar variety.

**Figure 3:**
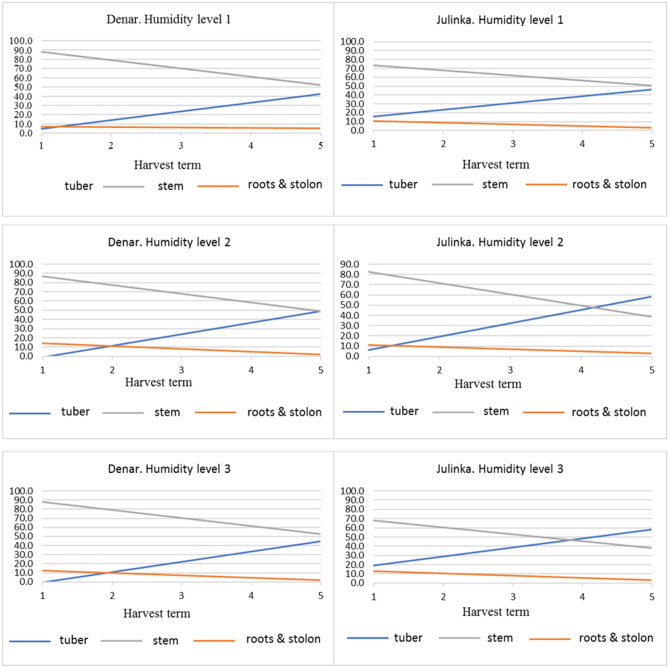
Potato biomass structure changes depending on humidity level and tuber harvest term (percentage).

Regardless of the humidity level and variety, the trends in the biomass yield structure were similar (Fig. 3). A downward trend from the 1st to 5th harvest period was shown for roots and stolons. This varied from 5 to 18% at the beginning of the study to 2–5% by the 5th period. It should be noted that under level 3, especially for the Denar variety, the percentage of roots and stolons was at a constant, low level. The percentage of stems with leaves decreased from 68–90% at the first harvest time to 40–55% at 5th. The dynamics of the decline in the share of stems and leaves was highest at humidity level 3. The tuber percentage was from 0 to 20% for the 1st period to 40–60% for the 5th period.

The Denar variety, regardless of the humidity level, was characterized by a greater share of stems and leaves. For the Julinka, the tuber percentage at the last harvest was at the same or higher than in the case of stems and leaves. At humidity levels 2 and 3, tubers accounted up to 60% of the harvested biomass.

The growth of stem and stolon biomass was noticeable at all stages of potato development (Table 2); greater dynamics were found in the growth of tuber mass (Fig. 3). Under level 3, the growth of the biomass of stems with leaves and stolons was slower than in level 2 of water was used.

### Water use efficiency

**Figure 4:**
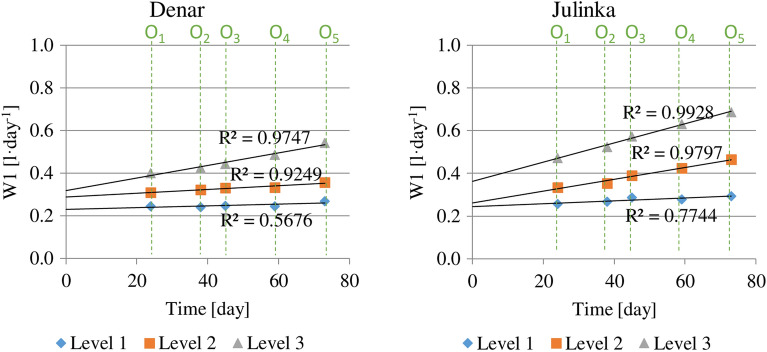
Average daily water consumption for potato varieties, at three soil humidity levels (1, 2, 3) and in each of five growing stages (O1), (O2), (O3), (O4), (O5).

Average daily doses of water used for the Denar and Julinka varieties in potato harvesting periods are shown in Fig. 4. The volume of water was determined each time for the corresponding level of humidity (1, 2 and 3). Based on the data obtained, a proportional increase in water consumption was found for both potato varieties. The most intensive increase in water consumption was noted at humidity level 3. The W index corresponding to the average daily dose of water calculated for the Denar variety varied from 0.40 l day^−1^ in the 1st period (O1) to 0.79 l day^−1^ in the 5th harvest period (O5), whereas for the Julinka it was from 0.49 l day^−1^ (O1) to 0.92 l day^−1^ (O5). The W values for the level 3 changed for the Denar variety from 0.23 l day^−1^ in (O1) to 0.38 l day^−1^ (O5), while for the Julinka from 0.28 l day^-1^ (O1) to 0.28 l day^-1^, respectively (O5). The difference in the intensity of water consumption increase for humidity levels was expressed by varying the values of simple directional coefficients approximating empirical data. The highest values of these coefficients were obtained for the humidity level 1. The directional coefficient for the Denar was 0.0077 day^−1^, and for the Julinka variety 0.009 day^−1^. For humidity level 3, these values are 4 and 6 times lower: 0.002 day^−1^ (Denar) and 0.0014 day^−1^ (Julinka), respectively.

**Figure 5:**
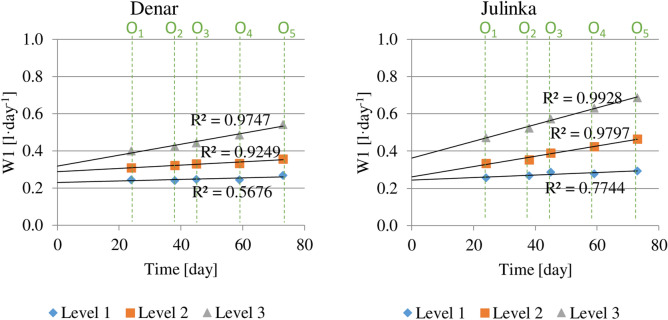
Average daily water consumption for potato varieties, at three soil humidity levels (1, 2, 3), cumulative calculation from potato planting.

The average daily water consumption throughout the growing season calculated from potato planting is shown in Fig. 5. The average daily water use was the highest for both varieties at humidity level 3. Index W1 for the Denar was 0.53 l day^−1^, while for Julinka was higher—0.70 l day^−1^. The water consumption for the humidity level 1 was about 2 times lower: for the Denar—0.27 l day^−1^ and for Julinka—0.29 l day^−1^.

Ahmadi et al. (2017)^37^ used various irrigation schedule strategies for water demand measurements at evapotranspiration. Water demand has been fully or partially satisfied in static and dynamic modes. The research presents dynamics of vapor pressure deficit (VPD) throughout the growing season. The value of VPD in the first days after planting the potato was about 0.5 kPa while in 70 days maximum value was noted (2.5 kPa), and at the end of the growing season (after 150 days) about 1.5 kPa. Due to the shorter potato growing season in present research, no decrease in water demand was noticed up to about 70 days and, as in the results of the research presented by Ahmadi et al. (2017)^37^, a steady increase in water demand was noted. Similar results were obtained by King et al. (2020)^38^ and the largest water deficit was found in the middle of vegetation, after 70–80 days after planting^35,39^.

**Figure 6:**
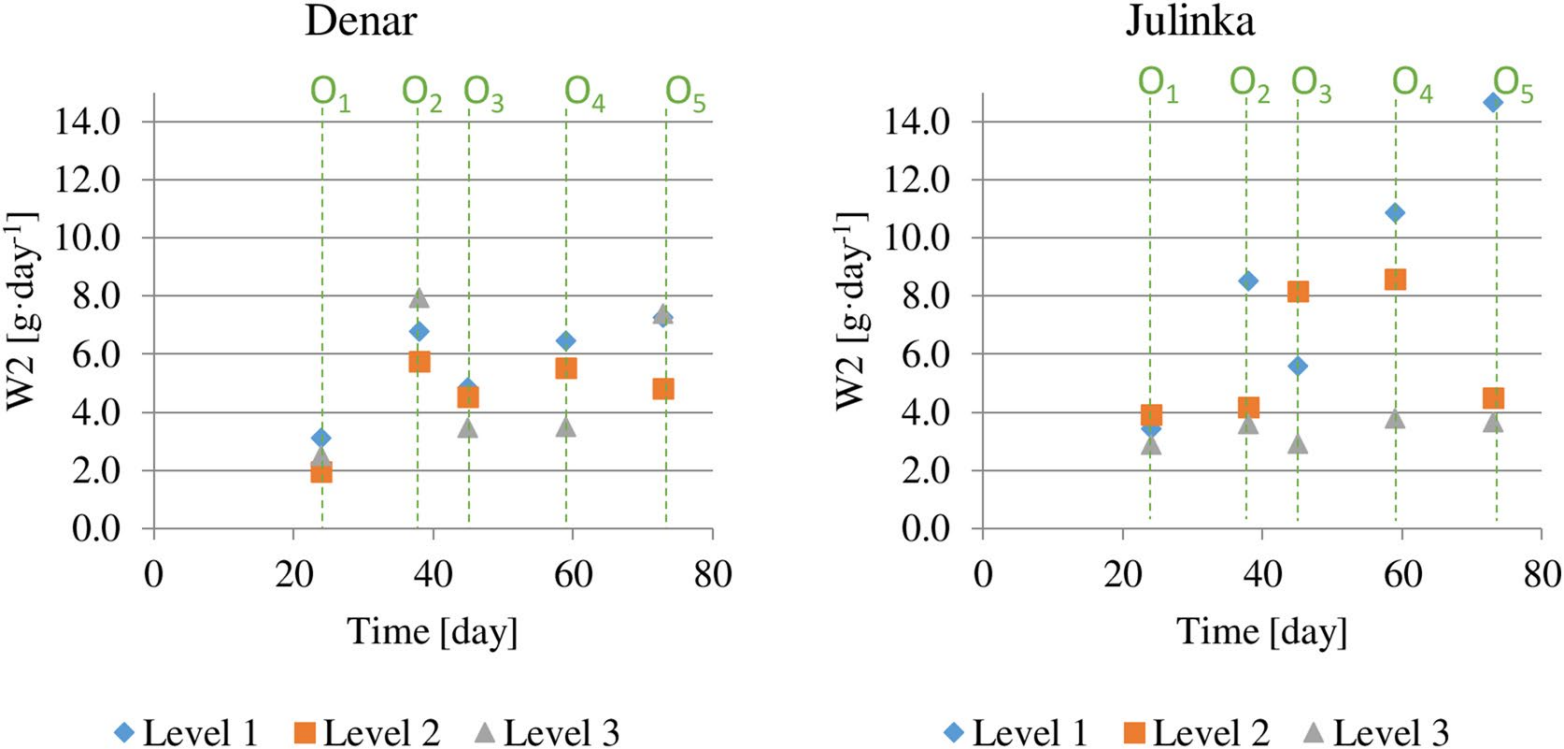
Average daily potato varieties tuber increase, at three soil humidity levels (1, 2, 3), in each of five potato growing stages (O1), (O2), (O3), (O4), (O5).

Values for average daily increase in potato tuber weight (index W2) in individual vegetation periods are presented below (Fig. 6). No approximation of functional models to empirical data is possible; hence, the conclusions are based on a description. In the 1st period, i.e. until day 24 (O1), tuber weight gains were smaller than in the other periods. Depending on the humidity level, these amounted to 2.0 to 3.5 g day^−1^ for the Denar variety, and 2.7 to 3.9 g day^−1^ for the Julinka. The differences for Denar were 1.5 g day^−1^ and for Julinka 1.2 g day^−1^. In the 2nd irrigation period (O2), average daily increase in potato tuber weight was the highest, from 5.9 g day^−1^ for level 2 to 7.9 g day^−1^ for level 3. Average daily tuber weight gain was 13% higher for level 1 than for level 2.

**Figure 7:**
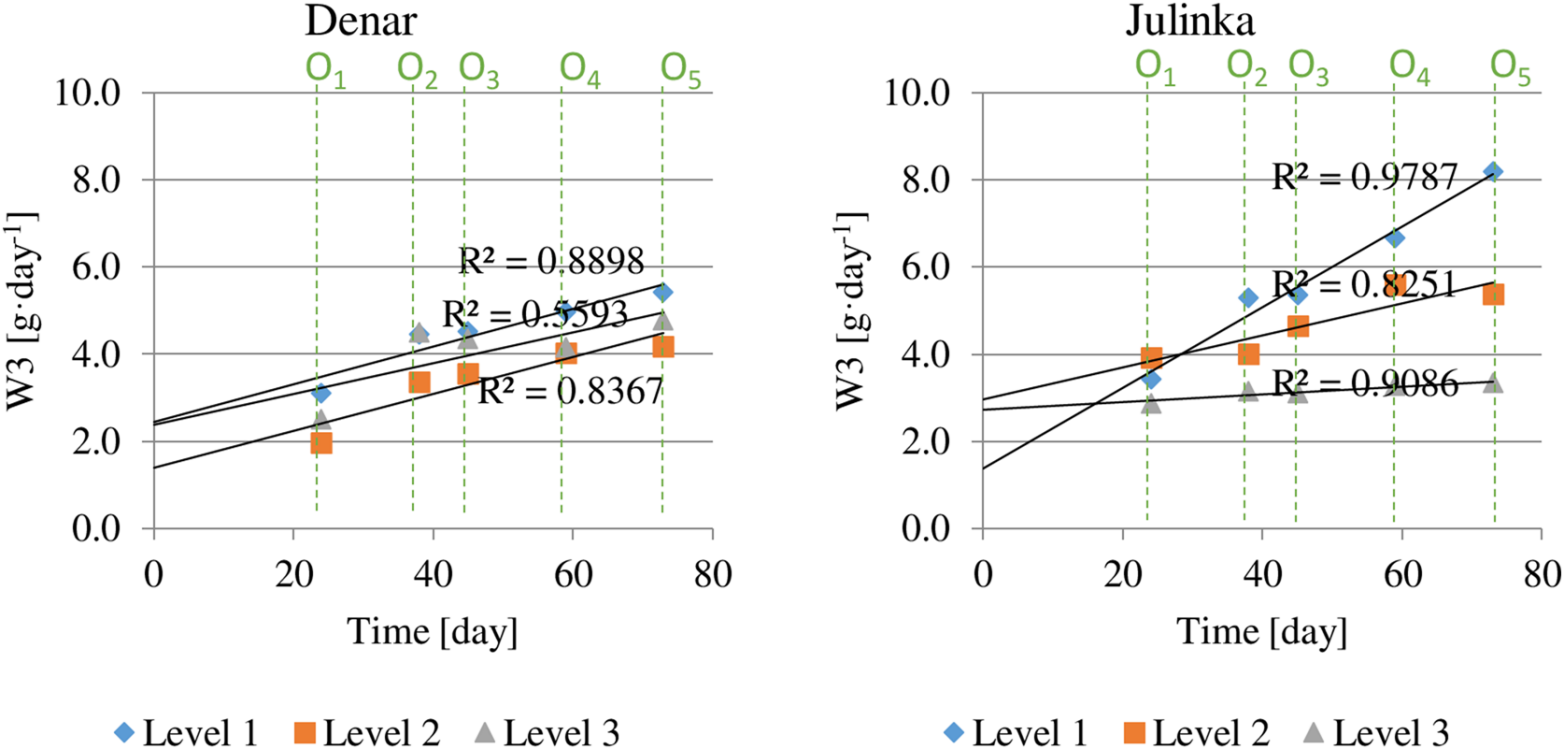
Average daily potato varieties tuber increase, at three soil humidity levels (1, 2, 3), cumulative calculation from potato planting.

The average daily weight gain of tubers of potato varieties (W3), calculated incrementally from the beginning of the experiment (Fig. 7). For the entire growing season, this indicator for the Denar variety was the highest for the humidity level 1st (5.7 g day^−1^), at the level 3rd (5.1 g day^−1^) and the lowest at the level 2nd (4.3 g day^−1^). The average daily weight gain of potato tubers of the Julinka was definitely highest for the first humidity level (8.1 g day^−1^).

**Figure 8:**
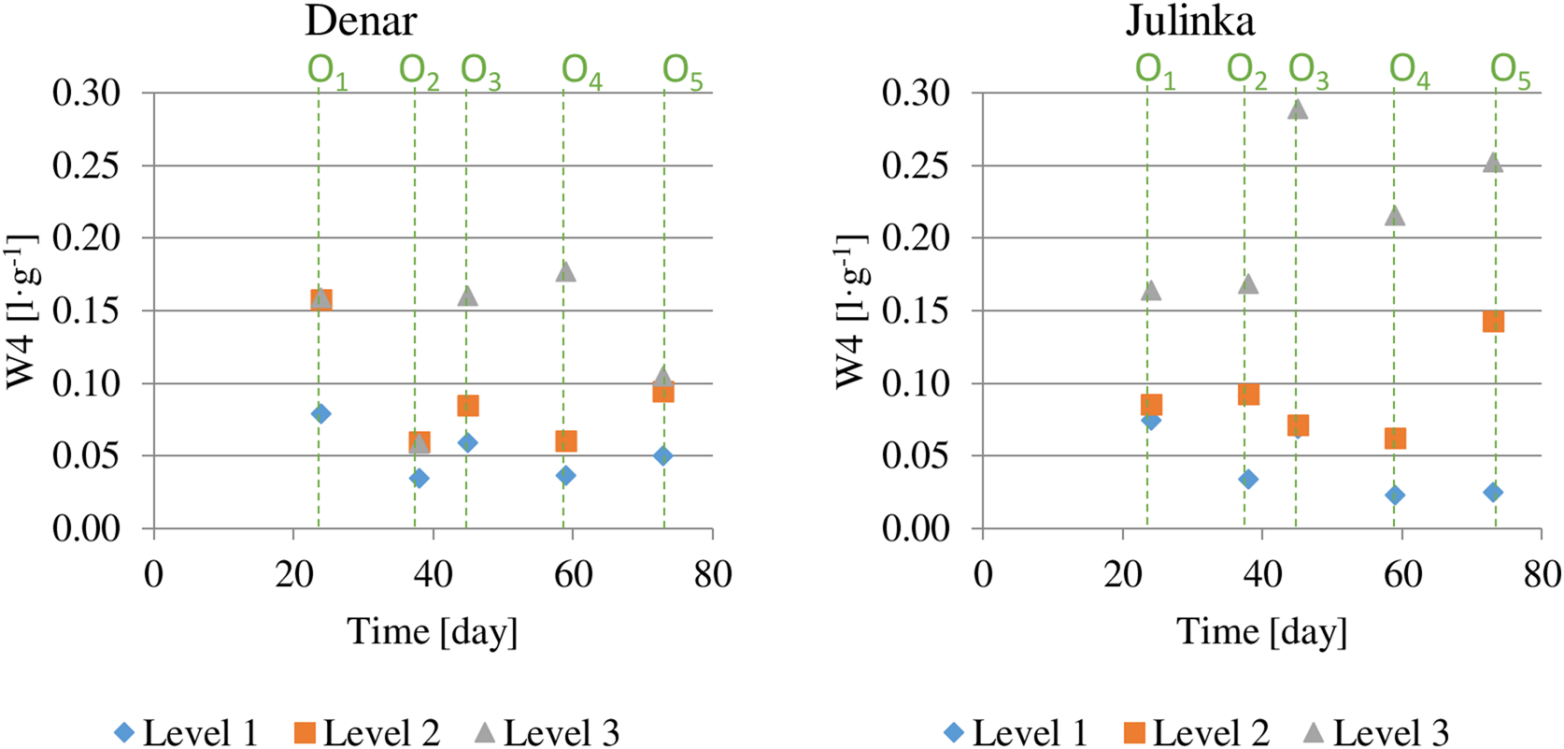
Ratio of average daily water consumption to average daily tuber mass increase dependent on three soil humidity levels (1), (2), (3), in each of five potato growing stages (O1), (O2), (O3), (O4), (O5).

The ratio of the average daily water consumption to the average weight gain of potato tuber (W4) for individual periods is given in Fig. 8. For humidity level 1 for Denar and Julinka varieties, the values decreased with the growing period of vegetation. In the period (O1), 0.079 l of water was used for the Denar variety and 0.075 l for the Julinka for an increase in potato tuber weight of 1 g. In the next stages of the growing season, this index ranged from 0.35 to 0.45 l g^−1^ for the Denar variety, for the Julinka it was definitely smaller and range from 0.25 to 0.34 l g^−1^. At humidity level 1, Julinka used less water than Denar to produce the same weight of tubers. At humidity level 2, the volume of water used at the beginning of growth was also the largest for the Denar variety (0.159 l g^−1^). This amount was two times higher than the volume at level 1. In subsequent periods, the indicator changed and ranged from 0.059 to 0.105 l g^−1^. For the Julinka variety, water consumption varied in individual periods from 0.085 to 0.113 l g^−1^ and showed no trend. At humidity level 3, Denar used the greatest amount of water, as compared to levels 1 and 2, and showing no trend. The Julinka variety used even more water at the same humidity level. This amount ranged from 0.164 to 0.298 l g^−1^ and, unlike in previous cases, it showed an upward trend with plant development.

Jovanovic et al. (2010)^40^ divided the potato growing season into five stages related to growth phases. There were no increases in the weight of leaves and stems, while the tuber weight, regardless of the irrigation method (PRD and FI), increased steadily. The weight of tubers in the last harvest, as compared to the first, increased five-fold. A similar relationship was obtained in the work of Shahnazari et al. (2007)^41^. This research also took account of different levels of humidity using the strategies of PRD and FI, also considering soil retention characteristics (pF curve). The research showed a clear steady increase in potato tuber weight in each harvest.

**Figure 9:**
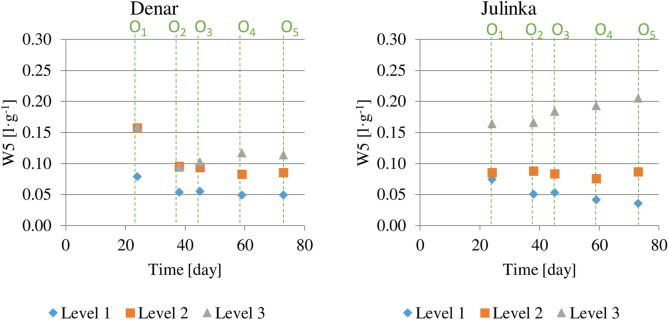
Ratio of average daily water consumption to average daily tuber mass increase dependent on three soil humidity levels (1), (2), (3), cumulative calculation from potato planting.

The ratio of the average daily water consumption to the average weight gain of potato tuber varieties calculated cumulatively from the planting (Fig. 9). The W5 value (0.114 l g^−1^) for the Denar variety at the end of the growing season was the highest for the 3rd humidity level and was about two times higher than at level 1. Water consumption efficiency for the Denar variety was the highest at humidity level 1. The sequence of W5 values is similar for the Julinka, with the difference that for the 3rd level it was 0.205 l g^−1^; i.e. six times higher than the indicator for level 1. Water consumption efficiency for the Julinka variety was definitely highest at humidity level 1.

Badr et al. (2010)^42^ analyzed the tuber yield, using two irrigation systems: surface and subsurface drip line. The total volume of water applied during the growing season was the differentiating factor. Results showed that as the volume of water applied during the growing season increased, the yield increased. When the subsurface line was used, applying 75 mm of water during the growing season, the total yield was approx. 27.5 t ha^−1^, and 32.5 t ha^−1^ for 325 mm. The effect of water amount on increase in yield was greater for the surface drip line. After applying 75 mm, the yield was 17.5 t ha^−1^, and 40 t ha^−1^ (for 325 mm). Similar results were obtained in the work of Linker et al. (2016)^43^. Regardless of the frequency, amount and total size of irrigation treatments, a proportional increase in the size of crops was observed with increasing doses of water.

Shahnazari et al. (2007)^41^ planned several harvest dates (H0–H4) throughout the entire growing season, analyzing the irrigation efficiency indicator (average WUE index’). Regardless of the irrigation technique, and taking into account, above all, the amount of water administered, the value of the average WUE index’ indicator was the highest in the period H2–H3, similar results were found in our own research.

## Conclusion

For the entire growing season, the average daily tuber mass gain was the highest for humidity level 1 (5.7 g day^−1^), and at humidity level 3–5.1 g day^−1^, while the smallest (4.3 g day^−1^) was recorded at humidity level 2. It was shown that maintaining excess water throughout the experiment was a stress factor for potato.

The highest water efficiency for both varieties was found at the assumed humidity level 1 and expressed for the dose of water consumed per 1 g of potato tubers. Efficiency was greater for Julinka than Denar and this amounted to 0.036 and 0.050 l per g (O5), respectively. Excessive humidity (level 3) caused a deterioration of the indicator. It was a maximum of 0.114 l per g for the Denar variety, and 0.205 l per g for the Julinka variety (O5). Maintaining humidity at a constant level of water deficit (pF 2.7) throughout the entire vegetation period ensured the most efficient water management.
